# Genome-wide scans provide evidence for positive selection of genes implicated in Lassa fever

**DOI:** 10.1098/rstb.2011.0299

**Published:** 2012-03-19

**Authors:** Kristian G. Andersen, Ilya Shylakhter, Shervin Tabrizi, Sharon R. Grossman, Christian T. Happi, Pardis C. Sabeti

**Affiliations:** 1 Department of Organismic and Evolutionary Biology, FAS Center for Systems Biology, Harvard University, Cambridge, MA 02138, USA; 2 Broad Institute, Cambridge, MA 02142, USA; 3 University of Ibadan, Ibadan, Oyo State, Nigeria

**Keywords:** Lassa fever, natural selection, positive selection, genome-wide scans, *LARGE*, interleukin 21

## Abstract

Rapidly evolving viruses and other pathogens can have an immense impact on human evolution as natural selection acts to increase the prevalence of genetic variants providing resistance to disease. With the emergence of large datasets of human genetic variation, we can search for signatures of natural selection in the human genome driven by such disease-causing microorganisms. Based on this approach, we have previously hypothesized that Lassa virus (LASV) may have been a driver of natural selection in West African populations where Lassa haemorrhagic fever is endemic. In this study, we provide further evidence for this notion. By applying tests for selection to genome-wide data from the International Haplotype Map Consortium and the 1000 Genomes Consortium, we demonstrate evidence for positive selection in *LARGE* and interleukin 21 (*IL21*), two genes implicated in LASV infectivity and immunity. We further localized the signals of selection, using the recently developed composite of multiple signals method, to introns and putative regulatory regions of those genes. Our results suggest that natural selection may have targeted variants giving rise to alternative splicing or differential gene expression of *LARGE* and *IL21*. Overall, our study supports the hypothesis that selective pressures imposed by LASV may have led to the emergence of particular alleles conferring resistance to Lassa fever, and opens up new avenues of research pursuit.

## 1. Introduction

Disease-causing pathogens are among the most intriguing forces shaping human evolution, as they have a tremendous impact on our genome and themselves evolve over time. They are also among the best-understood drivers of human evolution. In 1949, Haldane [1] made the observation that sickle cell anaemia, thalassaemias and other blood cell disorders were prominent in malaria-endemic regions of the world. He hypothesized that these disorders had become common in these regions through natural selection acting to increase the prevalence of traits that protect from malaria. ‘The Haldane hypothesis’ was confirmed in 1954 by A.C. Allison. Allison [2] demonstrated that the geographical distribution of the sickle cell mutation (Glu6Val) in haemoglobin B (*HbS*) correlated with malaria endemicity, was present only in Africa, and individuals carrying the sickle cell trait were resistant to malaria.

The confirmation of Haldane's malaria hypothesis provided an elegant first example of human adaptation, which gave strong support for Darwin's theory of natural selection proposed a century earlier. It also provided a clear demonstration of how natural selection can point us to biological mechanisms for resistance to infectious disease. As traits conferring resistance had arisen and spread in malaria endemic regions, the process generated striking and tractable differences between human populations—a signature of natural selection. Through natural selection, genetic variants that confer resistance to infectious diseases can spread through human populations over time, leaving such distinctive patterns in the human genome [3].

With emerging datasets of genomic variation in humans and pathogens, we can exploit the genetic signatures of natural selection towards identifying mechanisms of defence to many diseases [4]. The explosion in genotyping and high-throughput sequencing has made possible surveys of human genetic variation from multiple populations in large-scale collaborations such as the International Haplotype Map (HapMap) Consortium [5] and the 1000 Genomes (1000 G) Consortium [6]. The second phase of the HapMap project (HapMap II) allowed one of the first surveys of natural selection in the human genome, and examined 3.1 million single-nucleotide polymorphisms (SNPs) from 270 individuals from four populations: Yorubans from Nigeria in West Africa (YRI), Han Chinese from Beijing (CHB), Japanese from Tokyo (JPT) and European-ancestry individuals from Utah (CEU). The recently completed first phase of the 1000 G has enabled us now to look at genetic variation at every nucleotide in the human genome. For its initial release, 1000 G has provided approximately four-time coverage of whole genome sequencing from 179 individuals from the same four populations studied in the HapMap. The 1000 G dataset is currently expanding with more populations and individuals, but further quality control efforts need to be performed before it can be solely relied upon. Using these genotyping and sequencing datasets, several computational tools have been developed and applied to identify genes and regions in the human genome under positive selection (box 1) [3,7].

Box 1. **Signatures of natural selection detectable in genomic datasets. Most methods to detect natural selection fall into six categories of broadly defined signatures of positive selection.**

— *Function altering mutations (millions of years)*. When a protein is under strong selection, the number of non-synonymous (*D*_N_) to synonymous (*D*_S_) changes in its open reading frame may change dramatically. An excess of *D*_N_ suggests that positive selection has worked on the protein, whereas an excess of *D*_S_ suggests negative or purifying selection. Similarly, tests have been developed to identify an excess of potential function altering mutations in non-coding regions [8].
— *Reduction in genetic diversity around selected allele* (*less than 250 000 years*). As a variant under positive selection rises in frequency, ‘hitch-hiking’ nearby alleles increase in frequency as well. Such a ‘selective sweep’ leads to an overall decrease in diversity in the selected region with a simultaneous increase in the number of rare alleles as new SNPs are ‘born’ near the positively selected allele.
— *Increase in the frequency of derived alleles* (*less than 80 000 years*). When new alleles arise, they have a lower frequency than already present (ancestral) alleles. However, during a selective sweep, the selected allele as well as nearby neutral derived alleles will rapidly rise in frequency. A region with a high proportion of many derived alleles is therefore good evidence for positive selection having occurred in that part of the genome.
— *Increase in population differentiation* (*less than 75 000 years*) A particular allele may be beneficial in one population but not in another. In such a case, there will be a large difference between the frequency of the allele in one population versus the other.
— *Long-range haplotypes* (*less than 30 000 years*). Recombination during meiosis continuously breaks down associations between alleles on the same chromosomes. During a selective sweep, however, the selected variant rises quickly in frequency, leaving links with nearby alleles on the ancestral chromosome intact. This increase in ‘linkage disequilibrium’ leads to chromosomal regions where the haplotype is unusually long. This signature of positive selection can be measured using various haplotype-based tests, such as LRH [4], iHS [9] and XP-EHH [10].
— *Composite of multiple signals* (*CMS*) (*??? years*). While most of these tests have been successful in identifying regions under selection, in many cases, the individual genes or variants under selection remain obscured due to a lack of spatial resolution. Because strongly selected variants should contain many of the above five mentioned signatures, we have recently developed a ‘CMS’ method to improve spatial resolution up to 100-fold [11] allowing us to identify and localize specific variants under positive selection.

By examining evidence for natural selection in the HapMap data for YRI, we identified that one of the strongest signals of selection was at a 300 kb genomic region entirely within the gene *LARGE* [10]. The *LARGE* protein is a glycosylase that post-translationally modifies α-dystroglycan (α-DG), the cellular receptor for Lassa virus (LASV), and the modification has been shown to be critical for virus binding [12]. These results led us to the hypothesis that LASV protective alleles may have emerged and spread through West Africa, conferring resistance to severe disease from LASV infection.

Lassa haemorrhagic fever (LF) is a severe illness caused by LASV. It is endemic in West Africa and estimated to infect hundreds of thousands of individuals each year with thousands of deaths [13]. These numbers are probably underestimates, as most patients are never seen in hospital or are misdiagnosed with other febrile diseases such as malaria [14]. Past serological surveys point to its widespread impact, showing 21 per cent of Nigerians (approx. 30 million people) have had previous exposure to the virus [13], and exposure in parts of Sierra Leone and Guinea is above 50 per cent [13]. Therefore, LF is arguably one of the most neglected tropical diseases, given the number of people that it affects, its potential harm, and the unaddressed need for a better understanding of its complex biology.

In this study, we carried out a thorough examination of evidence for selection at genes biologically linked to LF, in order to pursue the hypothesis that these genes might be adaptations driving LF resistance in endemic areas. We confirmed the signal of selection at *LARGE* [10] and identified evidence of selection in West Africans for *IL21*—another gene biologically linked to LF. Using a recently developed computational approach, the composite of multiple signals (CMS), we narrowed the signals to within the first two introns of *LARGE* and to a cluster around *IL21* also containing *IL2* and *ADAD1*. We found that using either the HapMap II or 1000 G datasets gave similar results. In both cases, the top 10 high-scoring SNPs occur outside open reading frames (ORFs), suggesting that particular variants may have been selected based on their ability to affect gene regulation of *LARGE* and *IL21*. Using comparative genomics, we found that *LARGE* contains an unusually high proportion of SNPs in its ORF in humans but not in other species. It therefore appears that this gene, which we found to be under strict purifying selection in mammals, may experience diversifying selection in humans. Overall, this provides evidence for the hypothesis that selection pressures caused by LASV may have led to positive selection of particular alleles conferring resistance to LASV infection or disease.

## 2. Lassa fever

**Figure 1.**
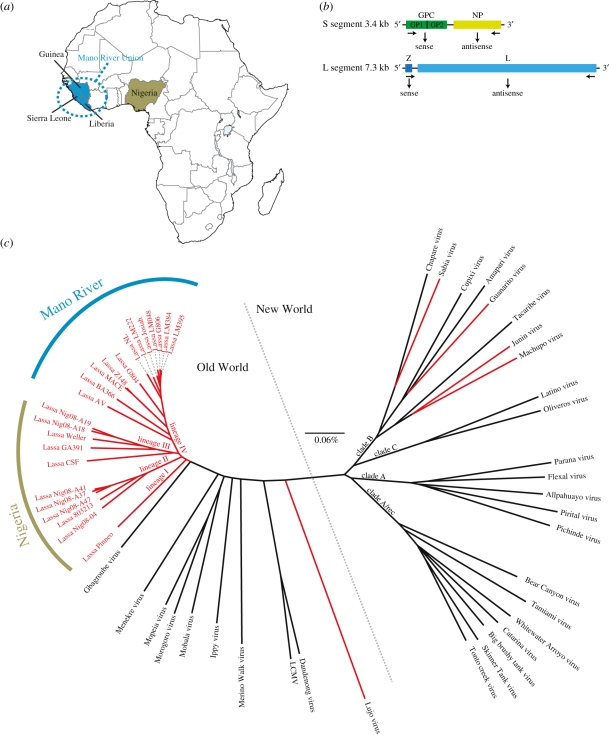
Lassa virus (LASV) is a highly divergent haemorrhagic fever-causing virus endemic to West Africa. (*a*) Map of Lassa haemorrhagic fever (LF) endemic countries. (*b*) The LASV genome consists of two RNA segments that encode four proteins using an ambisense strategy. The S segment codes for the nucleoprotein NP, as well the glycoprotein precursor GPC that is cleaved to the glycoproteins GP1 and GP2. The L segment contains the zinc-binding protein Z and the viral RNA-dependent RNA polymerase L. (*c*) LASV belongs to the highly divergent arenavirus family that is divided into ‘Old World’ arenaviruses mostly found in Africa and the ‘New World’ arenaviruses primarily found in South America. Representative full-length S segments from all known arenaviruses were aligned and a bootstrapped (1000 repetitions) phylogenetic tree was constructed using neighbour-joining [16]. Haemorrhagic fever-causing viruses are shown in red. Nucleotide divergence is indicated in the scale bar.

LF was first described in the town of Lassa in Northern Nigeria in 1969 [15]. It is endemic in West Africa with high disease prevalence in the Mano River Union countries, Sierra Leone, Guinea and Liberia as well as Nigeria (figure 1*a*), and sporadic outbreaks have been observed in neighbouring countries [13]. Its causal agent LASV is an enveloped, bisegmented single-stranded RNA virus (figure 1*b*) belonging to the large arenavirus family. While no accurate numbers for morbidity and fatality are available [17], it is believed that thousands of people die from the disease each year [13]. Despite the high rates of exposure in endemic regions and potential fatality, notably between 50 per cent and 90 per cent of West Africans infected show few to no symptoms of disease [13], suggesting that genetic factors of resistance may exist in the population.

While LF was not described until 1969, it is believed to be much older [18]. Sequencing studies of clinical isolates suggest that the virus originated in Nigeria, as most of the viral diversity can be found in this country [19] (figure 1*c*). Its natural reservoir *Mastomys natalensis* is probably the most common rodent to tropical Africa and rodent populations persistently infected with the virus have reached countries throughout West Africa [17]. They are commonly found near human settlements in rural areas, and are eaten as an important source of protein in some regions [20]. Before urbanization and domestication of plants and animals, ancient West Africans’ living and subsistence patterns would probably have created greater risk of disease than in most modern populations [21]. The likely antiquity of the disease and continual exposure to the rodent reservoir over generations are characteristics that make LASV a good candidate for selective pressure. Moreover, while today the case fatality is approximately 5 per cent in the general population, the fatality is much greater in pregnant women with foetal mortality nearly 100 per cent [22], creating a strong selective pressure acting on each generation. These findings—coupled with the high rates of observed disease resistance in West Africa, significant mortality and disability in affected individuals and sharing of α-DG as an entry receptor amongst arenaviruses [23]—suggest that LASV and LASV-like viruses serve as a likely source of strong selective pressure, driving genetic variants conferring disease resistance to high prevalence.

## 3. Genes implicated in Lassa fever are under positive selection in West Africa

**Figure 2.**
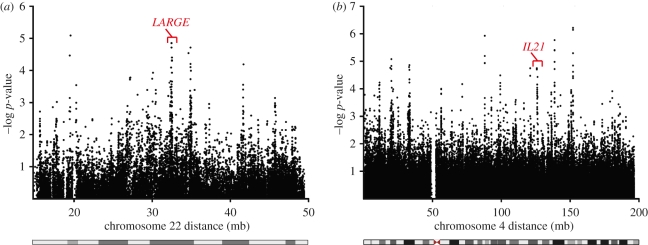
Chromosome-wide detection of positive selection at the *LARGE* and *IL21* loci in Yorubans from West Africa (YRI). (*a*,*b*) iHS scores were calculated from the HapMap II dataset and the −log *p*-values for the event that a SNP is under positive selection are shown [9].

Given the potential impact of a disease as severe as LF on human genome evolution, we decided to investigate genes involved in LF pathogenicity that show evidence of recent positive selection. Using the HapMap II dataset, we scanned the human genome using the long-range haplotype method iHS [9] and found evidence for positive selection at the *LARGE* locus on chromosome 22 (figure 2*a*) and *IL21* locus on chromosome four (figure 2*b*) in YRI. The *LARGE* gene has previously been identified using other long-range haplotype methods [10] and we found that the signal is consistently found as one of the top-scoring genes using a variety of selection methods and datasets (data not shown).

Both *LARGE* and *IL21* have a strong biological association with LASV infection and disease. *LARGE* is a glycosyltransferase that modifies the mucin domain of the LASV entry receptor α-DG. It also interacts with the N-terminal domain of α-DG and is required for the ability of LASV to infect cells [12]. Therefore, it is possible that polymorphisms in *LARGE* found in the West African population may confer direct protection against LASV infection by decreasing the ability of the virus to enter the cell.

IL21 is a member of the common γ-chain family of cytokines, which also includes IL2 and IL15 [24]. It is crucial for effective clearance of chronic infection with another member of the arenavirus family—lymphocytic choriomeningitis virus (LCMV) [25–27]. This virus shares α-DG receptor binding and much of its biology with LASV and is a very commonly used pathogen in immunological research [28]. Our data suggest that polymorphisms in the region surrounding *IL21* might have been positively selected in West Africa, potentially making individuals better able to cope with LASV infection.

## 4. Positive selection within *LARGE* localizes to the first two introns

Having identified a signal of selection at the *LARGE* locus, we used CMS [11] to further narrow down the signal. As true causal variants should display most signatures of positive selection (long haplotype, high-derived allele frequency and high population differentiation), we expected this test to give us a much better spatial resolution [11]. We used data from the HapMap II project (approx. three million SNPs) and from the initial phase of the 1000 G (approx. 15 million SNPs) and compared the results from both datasets.

**Figure 3.**
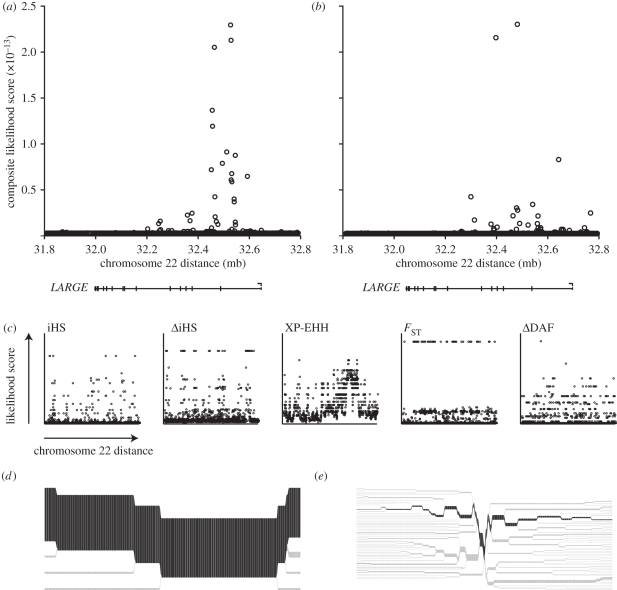
The signal of selection within *LARGE* localizes to the first two introns. (*a*,*b*) Composite of multiple signal-likelihood scores [11] were calculated in a 1 mb region of chromosome 22 using (*a*) HapMap II data (NCBI36/hg17 assembly) or (*b*) 1000 G data (NCBI36/hg18 assembly). (*c*) Likelihood scores of the individual tests that form the basis for CMS were plotted within the same region using 1000 G data. (*d*,*e*) Bifurcation diagrams [29] showing the extent of haplotype breakdown surrounding a putative selected allele at *LARGE* for the (*d*) derived and (*e*) ancestral allele in Yorubans from West Africa. The diagrams were created for the SNP with the highest value of iHS in the CMS top-scoring SNPs. The proposed ancestral (most abundant) haplotype on which the allele arose is shown in dark grey, whereas branch points are shown in light grey.

We found that using either dataset, the signal of positive selection localizes to the first two introns of *LARGE*. While the HapMap II analysis gave approximately 15 high-scoring SNPs (figure 3*a*), the 1000 G analysis narrowed that down to around five (figure 3*b*). Notably, all the five individual tests that form the basis for CMS [11] were able to pick up signals within *LARGE*, but with a much lower resolution (figure 3*c*). The fact that the selection signal is placed mainly within introns suggests that the selected allele of *LARGE* may be differentially regulated or alternatively spliced.

To better visualize the extent of long-range associations of the ancestral and derived alleles, we created haplotype bifurcation diagrams [4,29]. Here, the middle of the diagram displays the *LARGE* core haplotype. Going in either direction, it shows the breakdown of proximal and distal linkage disequilibrium, by branching off every time a new allele is present. The thickness of the lines corresponds to the number of samples with the indicated haplotype. As expected, the derived allele of *LARGE* displayed clear long-range associations to other neighbouring polymorphisms (figure 3*d*), a signal suggestive of the recent emergence of a young allele with an unusually high frequency in West Africa (30% frequency in YRI, 0% in CEU and CHB + JPT). In contrast, the ancestral allele of *LARGE* showed no such long-range associations, as is expected for an old allele (figure 3*e*).

## 5. Selection around *IL21* localizes to a region containing three different genes

**Figure 4.**
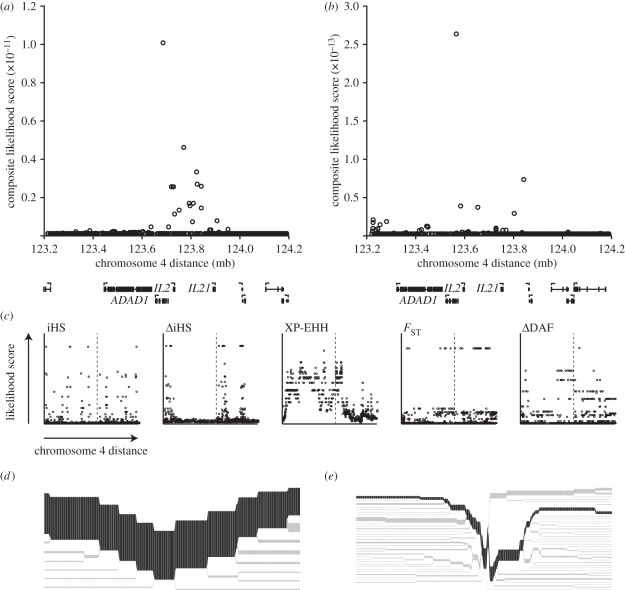
The signal of selection around the *IL21* locus. (*a*,*b*) Composite of multiple signal-likelihood scores [11] were calculated in a 1 mb region of chromosome 4 using (*a*) HapMap II data (NCBI36/hg17 assembly) or (*b*) 1000 G data (NCBI36/hg18 assembly). (*c*) Likelihood scores of the individual tests that form the basis for CMS were plotted within the same region using 1000 G data (dotted line, *IL21* locus). (*d*,*e*) Bifurcation diagrams [29] showing the extent of haplotype breakdown surrounding a putative selected allele at *IL21* for the (*d*) derived and (*e*) ancestral allele in Yorubans from West Africa. The diagrams were created for the SNP with the highest value of iHS in the CMS top-scoring SNPs. The proposed ancestral (most abundant) haplotype on which the allele arose is shown in dark grey, whereas branch points are shown in light grey.

We used CMS to localize the signal of selection observed near the *IL21* locus. Using HapMap II and 1000 G analysis, we found that the signal narrowed down to a 300 kb cluster on chromosome four containing the genes *ADAD1* and *IL2* in addition to *IL21* (figure 4*a*,*b*). Similar to that observed for *LARGE*, data from HapMap II or 1000 G gave comparable results, with 1000 G having fewer high-scoring SNPs. Again, CMS had much better power at localizing SNPs under selection than any of the five individual tests (figure 4*c*). As expected, the derived allele of *IL21* (59% frequency in YRI, 25% in CEU and 4% in CHB + JPT), unlike its ancestral counterpart, displayed long-range associations with nearby alleles when visualized in haplotype bifurcation diagrams (figure 4*d*,*e*).

The top-scoring SNPs by CMS fall outside any of the three genes' ORFs, suggesting that selection may have targeted variants that give rise to differential gene expression—either at individual genes, or over the whole cluster. Given the close spacing of the two common γ-chain cytokines *IL2* and *IL21*, it is interesting to speculate that selection may have targeted regulatory regions controlling the expression of both. Indeed, this potential appears to exist within these loci, as certain SNPs in this region have been implicated in an increased susceptibility to the autoimmune disease type I diabetes [30].

## 6. *Large* and *IL21* show different ancient patterns of evolution

**Figure 5.**
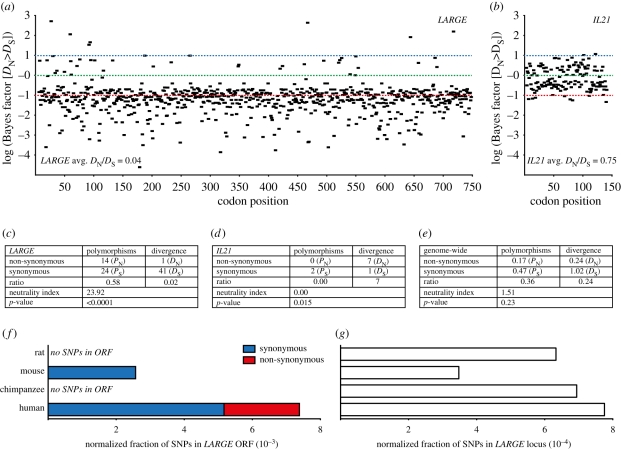
The open reading frames (ORFs) of *LARGE* and *IL21* show evidence of purifying and positive selection. (*a*,*b*) The ORFs from mammalian *LARGE* and *IL21* were codon-aligned and the ratio of non-synonymous (*D*_N_) to synonymous (*D*_S_) mutations were counted and the ratio between the two calculated. A log Bayes factor providing statistical support for *D*_N_ > *D*_S_ at individual sites was calculated using the random effects likelihood test implemented at the Datamonkey website [31]. Cutoff values for positive, neutral and purifying (negative) selection are marked on the diagrams in blue, green and red, respectively. (*c–e*) McDonald–Kreitman tests comparing the amount of polymorphisms in *LARGE* and *IL21* to that of the divergence in these genes between humans and macaques [32,33]. (*e*) Results were compared with the genome-wide values calculated from Bustamante *et al.* [34] (note that this comparison was between humans and chimpanzees). A neutrality index was calculated as (*P*_N_/*P*_S_)/(*D*_N_/*D*_S_). *p*-Values were calculated using a two-sided chi-squared test. Values below 0.05 were considered statistically significant. (*f*,*g*) The ORF of *LARGE* in humans has an unusually large number of SNPs compared with other species. (*f*) The numbers of synonymous and non-synonymous polymorphisms in the ORF of *LARGE* from human, chimpanzee, mouse and rat were retrieved from dbSNP and normalized to the total number of SNPs found in the *LARGE* locus from the respective species. (*g*) The fraction of SNPs found in the *LARGE* locus normalized to the total number of SNPs from the individual species.

Having identified signatures of recent positive natural selection in the genomic regions containing *LARGE* and *IL21*, we turned our attention to look for evidence of ancient natural selection within the ORFs of these two genes. CMS and haplotype-based methods allow for the detection of recent and ongoing selection in the human genome (within the last approx. 30 000 years) [3]. In contrast, methods to detect selection based on multiple species comparisons such as non-synonymous to synonymous (*D*_N_/*D*_S_) can elucidate evidence of natural selection as far back as the human split from other apes millions of years ago [3]. We codon-aligned the ORFs of the genes from at least 10 mammals and performed a *D*_N_/*D*_S_ analysis across the entire coding sequence. At individual sites, an excess of *D*_N_ over *D*_S_ is suggestive of positive selection, whereas a larger number of *D*_S_ over *D*_N_ is suggestive of purifying selection. Using the random effects likelihood and fixed effects likelihood tests incorporated in the HyPhy package on the Datamonkey website [31], we found that the *LARGE* gene has been under very strong purifying selection in mammals (figure 5*a*) and the gene is 100 per cent identical in humans and chimpanzees (data not shown). In contrast, *IL21* appears to have been under moderate positive selection in these species (figure 5*b*).

Next, we performed McDonald–Kreitman tests on the ORFs of *LARGE* and *IL21* to compare the level of genetic variation within human populations (polymorphisms) with that of genetic variation between species (divergence). For the purpose of minimizing the number of multiple mutations at individual sites, we limited our comparison to humans and macaques—two closely related species. However, we obtained very similar results when we compared humans with mice or rats (data not shown).

For *LARGE*, the ratio of non-synonymous to synonymous changes between species was significantly lower than the ratio of non-synonymous to synonymous polymorphisms (figure 5*c*; 0.02 versus 0.58; neutrality index: 23.92; *p* < 0.0001). This is vastly different from the 1 : 1 ratio (neutrality index: 1) expected under neutral conditions [32] and is unlike that observed for the genome as a whole (figure 5*e*). The very low proportion of non-synonymous substitutions between species suggests that the gene has been under strong purifying selection within the mammalian lineage. On the other hand, we identified an unusually high proportion of polymorphisms in the ORF of human *LARGE*. When we compared this with other polymorphism datasets in the Single Nucleotide Polymorphism Database (dbSNP), we found that the gene had substantially more polymorphisms in human populations than in rats, mice and chimpanzees (figure 5*f*,*g*)—even when corrected for the larger number of described human SNPs (figure 5*f*). While we found a significant number of both synonymous and non-synonymous SNPs in human *LARGE*, we observed only synonymous SNPs in mice and no SNPs at all in chimpanzees and rats. This, combined with our sliding-window analysis (figure 5*a*), suggests that whereas *LARGE* has been under strong purifying selection in the mammalian lineage, it may be more recently under continuous diversifying selection in humans.

For *IL21*, the scenario is the opposite of that observed for *LARGE*. For this gene, the ratio of non-synonymous to synonymous changes between species was significantly greater than the ratio of non-synonymous to synonymous polymorphisms (figure 5*d*; 7 versus 0; neutrality index: 0.00; *p*-value: 0.015). This is different from the ratio expected under neutral conditions and is also unlike the genome-wide observation (figure 5*e*). Rather, this suggests that *IL21* has undergone positive selection in the mammalian lineage, whereas its ORF appears fixed in the human population.

## 7. Hypotheses for how variants of *LARGE* and *IL21* may confer selective advantages

The molecular mechanisms behind positive selection can target multiple biological pathways (table 1). SNPs can lead to either non-synonymous, synonymous or non-coding changes that may alter protein function or expression. Because most of the high-scoring SNPs in *LARGE* and *IL21* fall outside the ORFs, positively selected variants of these genes may lead to regulatory changes such as differential gene expression. Such a change can result in a global change in gene expression leading to a decreased or increased expression of the genes. Alternatively, the changes in gene expression may only affect particular cellular subsets or tissues. For example, because dendritic cells express high levels of α-DG and appear to be direct targets of LASV infection [35], a difference in gene expression of *LARGE* in these cells may lead to under or over glycosylation of α-DG, interfering with LASV infectivity [28]. The most attractive hypothesis involves SNPs leading to decreased expression of *LARGE* and therefore a reduced susceptibility to LASV infection via a direct action on α-DG [12]. However, it has also been shown that LASV itself downregulates *LARGE*/α-DG—probably in order to bud off from infected cells [36]. Another possible scenario would therefore involve SNPs resulting in an increased expression of *LARGE* and the inability of the virus to leave infected cells. Given the localization of the signal to within the first two introns of *LARGE*, positive selection may also have given rise to alternative splice variants of this gene interfering with its function.

**Table 1.** Positive selection at individual loci can result in a multitude of different molecular, cellular and biological changes.

(*a*) target of selection		
resistance to disease	resistance to infection	
no illness does not progress to severe stage antibodies expected to be present	no infection lowered infection antibodies expected to be absent or low	
(*b*) biological effects		
direct/cell autonomous	indirect/systemic	

e.g. decreased infectivity e.g. *LARGE*	e.g. increased immunity e.g. *IL21*	
(*c*) mechanisms		
	gene expression	protein altering
	
where?	regulatory regions	within the protein
	promoter, introns, ORF and regulatory non-synonymous and synonymous	ORF non-synonymous
consequences	differential gene expression	different protein function
	systemic change in gene expression tissue-specific changes cell-specific changes	enhanced/decreased protein activity new protein function loss of protein function
laboratory tests	standard and genome-wide tests	custom-made assays
	gene-specific qPCR promoter-specific reporter assays RNAseq microarray	biochemical assays, e.g. glycosylation cellular assays, e.g. infectivity testing changes in signalling pathways, e.g. reporter assays for NF-κB activity

No non-synonymous changes have been observed in the ORF of *IL21*, and our selection peak near this gene lies together with *IL2* and *ADAD1*. The most likely scenario is therefore that SNPs under positive selection may have targeted regulatory elements causing a change in gene expression. Unlike *LARGE*, a potential protective role of *IL21* in LF would probably be more systemic and not specific to a single pathogen. Because this cytokine has been shown to be involved in the clearance of the arenavirus LCMV [25–27], it is possible that certain SNPs leading to increased expression of *IL21* would have a protective role in LF progression. This could ultimately results in enhanced immunity against LASV and improved ability of the immune system to cope with the infection. However, as with *LARGE* the picture may be more complicated. It is currently unknown what causes LF to progress from severe to fatal disease, but a likely explanation is that the human immune system 'overreacts’, generating hyper-active JAK/STAT and NF-*κ*B signalling pathways. The resulting ‘cytokine storm’ may ultimately be the reason for a fatal outcome [37]. Decreased expression of *IL21*, and potentially the closely linked *IL2*, may therefore have protective effects by halting the progression of LF from severe to fatal disease.

## 8. Future directions

With ever-increasing numbers of large-scale datasets and powerful computational methods to detect signals of natural selection, the pursuit of evolutionary adaptations in humans has moved from testing of specific observations such as sickle cell and malaria resistance to a hypothesis-generating process. Now with many candidates to pursue and better tools to pinpoint individual SNPs under selection, the step from hypothesis generating to hypothesis testing is as opportune as ever. We have identified two genes biologically linked to LF pathogenesis that make several testable predictions, and we have initiated collaborations across West Africa in order to test them. In particular, we would expect to find extreme correlations between the absence or presence of protective alleles when comparing the genotypes of LF patients and unrelated controls. Given that a particular variant might confer protection from LF, one would expect the frequency of this allele to be absent or much lower in LF patients when compared with the general population. Combining this with serological assays to look for the presence of previous exposure to LASV should help us get a much clearer picture of the complex biology of this devastating disease.

Comparing results found in the YRI of West Africa to other more closely related African populations, but experiencing different selective pressures, should also prove productive. Indeed, phase III of the HapMap generated genome-wide data for two East African populations, the Misai and Luo of Kenya, showing that those African populations outside LF endemic areas do not carry the selected haplotype of *LARGE*, whereas the West African population YRI does. Similarly, the frequency of the selected haplotype of *IL21* is significantly higher in YRI than in these other two populations. However, more extensive surveys are required in order to firmly establish this and we are setting out to examine other West African populations where LF is and is not endemic to further investigate the presence or absence of selected haplotypes of *LARGE* and *IL21*.

Finally, for both *LARGE* and *IL21* our studies suggest that the most likely target of selection has been differential gene regulation. This needs to be confirmed through extensive experimental studies, such as qPCR profiles for LF patients and controls combined with reporter assays and biochemical tests *in vitro*. Combined, agnostic computational methods such as CMS and rigorous reductionist hypothesis testing in the laboratory should enable us to take the ever-increasing list of natural selection candidates to validated examples of evolution in the human species.
